# Ecotourism design and plant protection based on sensor network

**DOI:** 10.3389/fpls.2022.993838

**Published:** 2022-09-12

**Authors:** Jiang Zhu, JinChun Sun

**Affiliations:** ^1^School of Management, Xi’an Jiaotong University, Xi’an, China; ^2^Department of Design, Taiyuan Normal University, Taiyuan, China

**Keywords:** sensor network, ecotourism design, plant protection, forest park, conservation area

## Abstract

National Forest Park is an important place for the public to carry out forest recreation activities and recognize natural habitats. With the popularization of forest tourism and the increase of forest recreational activities, the pressure on forest habitats has increased. The development of national forest parks is accompanied by opportunities and challenges. The main purpose of this paper is to analyze and study the impact of ecotourism design on plant protection based on sensor network technology. This paper analyzes the impact of tourism on the ecological environment, establishes an ecological environment monitoring system and an ecological tourism resource evaluation system, and studies the functional division of forest parks. Experimental research shows that, as a strictly protected area, the ecological conservation area basically does not conduct scenic spot development and resource mining, nor is it open to tourists. The total area is 852.92 ha, accounting for 22.31% of the total area of the forest park, allowing the ecology of the ecological conservation area to achieve sustainable and healthy development.

## Introduction

With the development of science and technology and the leap of economy, the integration of agriculture and eco-tourism is developing rapidly, and the agro-eco-tourism industry has promoted the transformation of agricultural industrial structure and the improvement of efficiency, and has been flourishing in all parts of my country. The basic characteristics of agricultural ecotourism include dependence on ecological agriculture, taking ecological environment protection as the core, combining with rural culture, and extensive participation in tourism activities. At the same time, due to the insufficient understanding of the role of plant protection in tourism, the surrounding environment has deteriorated and the risk of large-scale outbreaks of pests and diseases has increased. From the academic point of view, ecotourism mainly focuses on planning, economy and ecology, but there are few theoretical studies on the role of plant protection on ecotourism (Awasthi and Bollas, 2020; Manoj et al., 2020). The construction of ecological agriculture has laid a good material foundation and pollution-free security for the development of tourism. The establishment of green agricultural product bases in tourist attractions and the adoption of science and technology to plant crops are not only conducive to the promotion and application of agricultural technology, but also provide green food for tourists. Tourism and eco agriculture are combined, and they complement each other and promote each other, so as to realize the ecological, social and economic benefits of agricultural eco-tourism.

In related research, Sambo et al. (2020) mentioned that wireless underground sensor network (WUSN) faces the problem of wireless underground communication (WUC), which greatly attenuates ground signals. Wireless sensor network unit includes data acquisition unit, data transmission unit, data processing unit, and energy supply unit. At the end of it is a sensor that can sense and inspect the external world. His sensors communicate wirelessly. From this, a WUSN path loss for precision agriculture is proposed, called WUSN-PLM. To achieve this, the proposed model is based on an accurate prediction of the complex permittivity (CDC). Martin et al. (2021) proposed an algorithm to map direct normal irradiance (DNI) in thermal solar power plants using a mobile robotic sensor network (RSN). The algorithm selects measurement points and assigns RSN accordingly for dynamic estimation of DNI. Sensor network realizes three functions of data acquisition, processing and transmission. Together with communication technology and computer technology, it constitutes the three pillars of information technology. The performance of the algorithm is evaluated using a generic thermal solar power plant with a fleet as a simulated case study.

Based on sensor network technology, this paper analyzes and studies the impact of ecotourism design on plant protection. This paper first introduces the meaning of ecotourism, and analyzes the four principles of ecological planning, namely, the principle of ecological protection, the principle of overall optimization, the principle of adapting to local conditions, and the principle of landscape heterogeneity. The impact of water bodies, soil, plants, and wild animals; then, the data processing technology is analyzed, the ecological environment monitoring system and the ecotourism resource evaluation system are established, and the comprehensive ecological characteristics are analyzed. Note that For the development and ecological protection of tourism resources, combined with the comprehensive improvement of key river basins and regional environment, the environmental management of tourist areas has been strengthened, the environmental impact assessment has been carried out for the planning, development and construction projects of some tourist areas, the pollution prevention and control efforts have been strengthened, and a number of polluting enterprises in scenic tourist areas have been shut down, relocated and treated within a time limit (Palani et al., 2020).

## Design research

### The meaning of ecotourism

The meaning of ecotourism has been constantly changing, and the process of changing the definition of forest park is similar, but the emphasis is still different. Tourism, with distinctive ecological environment as its main landscape, takes sustainable development as the concept, takes the protection of ecological environment as the premise, takes the harmonious development between man and nature as the criterion, and relies on a good natural ecological environment and a unique human ecosystem. The key points include ecotourism tourist motivation, ecotourism resource level, socioeconomic capacity of the region, and ecological capacity of forest parks. Because ecotourism is different from other forms of tourism, it appeared in a later period, so it is not very familiar to the public, and it is easy to be confused with other concepts such as mass tourism, nature tourism, and sustainable development tourism that have appeared in research for a long time (Baghaee et al., 2020; Venkataravanappa et al., 2020).

Distinguishing analysis of similar concepts and categories:

One is the difference between ecotourism and mass tourism. Compared with ecotourism, mass tourism emerged earlier, and ecotourism is a derivative of mass tourism. Because mass tourism is an early concept and form of tourism, its connotation does not include the protection of natural environment and ecological resources, the threshold for development and introduction is low, and it is easy to develop and implement, but there are also problems of damage and negative impact on forest resources. The first meaning of mass tourism refers to that the range of participants in tourism activities has been extended to ordinary working people. The second meaning is that modern tourism activities began to form a mass tourism model represented by organized group package tourism, and formed a dominant tourism form among the general public. In terms of planning management, development goals, and stakeholders, ecotourism prioritizes resource protection and avoids the sacrifice of forest resources. Therefore, it is quite different from mass tourism.

The second is the difference between ecotourism and nature tourism. The difference between the two is small, the main reason is that both rely on natural resources and are closely related to animal and plant resources, as well as physical resources such as mountains and rivers. Natural tourism takes natural resources as the core of development power. Compared with mass tourism, it is more inclined to be close to natural resources, but it also lacks the popularization of environmental protection awareness. Therefore, tourism activities will still have an impact on natural resources. On the basis of natural tourism, ecotourism emphasizes the protection of the natural environment and ecological resources, from people-oriented to ecological resources-oriented, and realizes the value of ecotourism through the interaction between people and ecological resources in the natural environment. The basic principles of people-oriented management include: paying attention to people’s needs, encouraging employees, cultivating employees, and people-centered organization design.

Among these three tourism concepts, the origin of sustainable tourism comes from ecotourism. From the perspective of sustainable development, sustainable tourism is a persistent and continuous tourism behavior. The difference between sustainable development tourism and ecotourism is that sustainable development tourism contains more abundant concepts. There are various forms and processes of sustainable development tourism, and it is not limited to ecotourism. The main connotation of sustainable tourism includes improving people’s understanding of the environmental and economic impact of tourism, strengthening people’s ecological awareness, promoting the fair development of tourism, improving the quality of life in tourism reception areas, providing high-quality tourism services to tourists, and protecting the environmental quality on which future tourism development depends.

### Principles of ecological planning

#### Principles of ecological protection

The ecological elements of the ecosystem play an important role in the stability of the resource utilization of the forest park. Therefore, in the planning, it is necessary to respect and conform to nature, and on this basis, adhere to the principle of ecological security to provide the maximum survival for the biological groups in the forest park. It is necessary to improve the ability to protect ecological resources, maintain the connectivity between various landscapes, and create a living environment space suitable for biological habitats. Ecological security has the characteristics of integrity, irreversibility and long-term. Specifically, ecological security is a state of human living environment or human ecological conditions. Ecological security is a dynamic concept. Ecological security emphasizes people-oriented, and maintaining ecological security requires costs. Focusing on the comprehensive protection of landscape resources, a targeted forest park planning scheme is formulated, and ecological protection and forest tourism are always positioned as the primary functions of the park, and development is carried out on the basis of sustainable development (Blake et al., 2021).

#### The overall optimization principle

The principle of overall optimization should be considered, so that the internal functional division of the forest park can be adapted to the surrounding environment, and the symbiosis and prosperity of the forest park and the surrounding environment should be ensured through overall protection and system optimization. The attributes and functional properties of each component in the park are different, but the overall style and form should be unified, and the overall characteristics and image characteristics of the forest park should be maximized through analysis, and each element must be balanced in the ecological environment. We can’t ignore one and lose the other, take the overall optimization as an important organic part of ecotourism, and strive to create an ecologically harmonious forest ecosystem.

#### The principle of adjusting measures to local conditions

When planning and designing suburban forests, it is necessary to respect the *status quo* of the natural, cultural and economic conditions of the region, and carry out practical designs based on research and analysis of superior planning, so as to highlight the characteristics of suburban forest parks. It is necessary to focus on ecological conservation and cultural protection, and design various places for forest tours, popular science education, and outdoor recreation according to local conditions. The construction of suburban forest parks is to protect the original ecological environment and develop its unique landscape on this basis. Select native plants, give full play to climatic characteristics, properly use exotic plants and cultivate new high-quality tree species to form a multi-layer mixed structure of trees, shrubs, grasses, and ground cover. Create infrastructures such as water landscapes, buildings, and sketches that are rich in local characteristics and forest park orientation, so that various landscape spaces can cooperate with each other to form a forest tourism environment with changing scenery and increase the fun of the tour. The economic characteristics of suburban forestry include the duality of suburban forestry, the diversity of suburban forestry production, and the economy of suburban forestry. Among them, the economy of suburban forestry is reflected as follows: on the one hand, facing the vast cities, suburban forestry has fast information, wide product sales and broad market prospects; On the other hand, the suburban forestry cannot be completed like the urban garden department, and the construction funds are allocated by the state to solve the construction of suburban forestry.

#### The principle of landscape heterogeneity

Landscape heterogeneity refers to the variability of landscape elements in the landscape system, which is beneficial to the division of the spatial pattern of the ecological environment and to improve patch heterogeneity and complexity. The degree of landscape heterogeneity is related to the biodiversity of the landscape. In the construction, the existing landscape heterogeneity should be used and strengthened, the vertical landscape design should be enriched, the aquatic plant habitat system should be established, and the ecological balance should be maintained. Strengthen the protection of ecologically sensitive areas, provide a heterogeneous living environment for the creatures in the park, strengthen the construction of landscape heterogeneity, and create a heterogeneous tourism space for tourists.

### The impact of tourism on the ecological environment of forest parks

The impact of tourism activities on the ecological environment is usually reflected in specific locations in tourist areas. Because the locations of natural landscapes and cultural attractions that attract tourists are relatively fixed, they account for a small proportion of the total area. Among them, the impact of tourism activities on vegetation and soil is relatively concentrated, which are fixed components of the ecosystem, while the impact on water bodies and wild animals is relatively scattered (Alkhalifa and Almogren, 2020; Khodadadiangostar et al., 2020).

#### Impact on water bodies

The impact of tourism activities on water bodies is mainly reflected in the quality of water quality, such as: eutrophication of water bodies, discarding of domestic waste, mass reproduction of planktonic algae, and water pollution caused by discarded suspended solids. Water pollution will directly affect the death and accumulation of aquatic animals and plants. The accumulation of pollutants causes harmful gases to be produced in water bodies. The human excrement produced by the residents living in the forest park also greatly pollutes the water quality, and the hotels, shopping streets, restaurants and other tourist service places opened near the water resources will also produce non-degradable waste when entertaining tourists. It takes a long time to purify the wastes to remove the pollution. If these pollutants are not treated scientifically and properly, it will cause water pollution in the forest park and surrounding water bodies. In the surface fresh water system, phosphate is usually the limiting factor of plant growth, while in the seawater system, ammonia nitrogen and nitrate are often the limiting factors of plant growth and total production. The substances that lead to eutrophication are often the nutrients with limited content in these water systems. Accordingly, the prevention and control measures include controlling the input of exogenous nutrients and reducing the load of endogenous nutrients.

Although the water body has a self-purification function and has a certain ability to withstand external influences, the pollution caused by the short-term is not significant, but after a long period of time, when the pollutants accumulate to a certain level, the problem will become prominent. The aquatic ecosystem has a dynamic equilibrium system, and usually all kinds of organisms in the aquatic ecological environment are in the trend of mutual balance. However, when various tourism activities follow, the water ecological balance will be broken, and the linkage of the entire ecological chain will begin to fail, which will reduce the stable development of aquatic organisms and the protection of diversity, will cause more serious ecological damage.

#### Effects on soil and plants

Because the growth of vegetation is inseparable from the soil, tourism activities, especially the impact of trampling on the soil, will indirectly affect the growth of vegetation. Vehicles roll on the soil, causing the soil to be compacted, or tourists trampling on the lawn, causing damage to the vegetation surface, severe surface exposure and serious soil erosion, which affect the growth and vitality of trees, which in turn has a series of cyclic effects on soil and vegetation. It is also related to the location of tourist routes and scenic spots. For scenic spots that are used more frequently, the scope of influence will expand with the extension of usage time and the increase of tourists’ demand for space.

The most direct consequences of tourism activities on soil and plants are: the reduction of surface vegetation coverage and the reduction of species diversity, which in the long run will affect the ecosystem of suburban forest parks.

#### Impact on wild animals

Tourism activities create a stressful environment for animals, and noise pollution from development and tourism activities has the greatest impact on wildlife. When animals live in an environment that exceeds the normal volume for a long time, they will become more and more timid and unresponsive. When encountering a moving vehicle or an advancing ship, they cannot avoid danger in time, resulting in an increase in accidental mortality, and even put some endangered animals on the verge of extinction. The trampling of natural ground cover by tourists and the uncontrolled picking of flowers and fruits of plants will also have a negative impact on the animal habitat in the forest park, which limits the living space and activity range of animals to a large extent. At the same time, hunting and hunting activities have a great impact on wild animals. The endless hunting activities of humans have led to a sharp decrease in the number of wild animals. The destruction of the overall ecological environment also has a certain impact on the number of wild insects (Nobar et al., 2020; Younus et al., 2020).

### Algorithm research

After the forest data collection is completed through the image, according to the characteristics of the Rikola hyperspectral remote control data, on the basis of completing the initial quality assessment of the image, the hyperspectral image is reproduced in the system and process. Compared with the traditional low spectral resolution remote sensing technology, hyperspectral remote sensing provides a wider application in earth observation and environmental investigation, which is mainly reflected in the following aspects: the resolution and recognition ability of ground objects is greatly improved, the imaging channel is greatly increased, and the influence of other interference factors is largely suppressed in the spectral space.

#### System calibration

In the process of acquiring hyperspectral images by the UAV platform, there will inevitably be systematic errors due to the limitations of the instrument itself and the measurement method, and such errors need to be corrected.

Vegetation hyperspectral images represent ground object information by pixel brightness value (DN value), but due to the influence of systematic errors, DN value cannot truly reflect the spectral properties of ground objects, and it is necessary to use measurement information representing specific signals obtained during the testing process. Convert the DN value of the original image to the reflectivity of ground objects, the formula is as (1).


(1) $$\rho_{t}=\frac{D\,N_{t}-D\,N_{1}}{D\,N_{2}-D\,N_{1}}\,\left(\rho_{2}-\rho_{1}\right)+\rho_{1}$$

In the formula, ρt and DN_*t*_ are the reflectivity and DN value of the original image target pixel, respectively, ρ_1_ and ρ_2_ are the reflectivity of different calibration oil cloths, respectively, DN_1_ and DN_2_ are the DN values of different calibration oil cloths, respectively. The main application of traditional imaging remote sensing technology is qualitative analysis, and the accuracy of some quantitative analysis results is not ideal. This is obviously due to the limitations of the spectrum and spatial resolution of the imaging sensor, the interference of the atmosphere and soil background, etc., which greatly suppresses the influence of other interference factors in the spectral space, which is of great help to improve the accuracy of quantitative analysis results.

#### Post-processing correction

The UAV images obtained by the frame-based imaging method in the flight experiment in this study are affected by factors such as the imaging principle and the environment. There are subtle differences in position and attitude among the 45-band images, resulting in the generated hyperspectral cubes with different bands in each band. Cannot completely overlap, we used the Coregister folder tool of Pix4D software for band registration. The time for a drone to fly once is about 20–30 min. Affected by changes in sunlight conditions, there will be gradient differences in the radiance between different flight zones, and the image will often show uneven color and brightness. Using equations (2) and (3) can effectively correct the irradiance to the normal level.


(2) $$L_{j\,c}\,{\left(\lambda \right)}_{a\,t\,\_\,s\,e\,n\,s\,o\,r}=L_{j}\,{\left(\lambda \right)}_{a\,t_{s\,e\,n\,s\,o\,r}}^{*}\,C_{j}\,\left(\lambda \right)$$

(3) $$C_{j}\,\left(\lambda \right)=E_{j}\,\left(\lambda \right)/E_{r\,e\,f}\,\left(\lambda \right)$$

Among them: L_*jc*_(λ)_*at_sensor*_ represents the image after irradiance consistency correction; L_*jc*_(λ)_*at_sensor*_ represents the jth original image; C_*j*_(λ) represents the multiplicative correction factor of j; E_*j*_(λ) represents the record of j Irradiance value; C_*ref*_(λ) represents the irradiance value of the reference image. Note that image gradient can regard image as a two-dimensional discrete function, and image gradient is actually the derivation of this two-dimensional discrete function. Image edge is generally realized by gradient operation of image.

## Experimental study

### Data processing technology

Using ArcGIS and ENVI software to preprocess the collected basic data, the main technical tools involved are as follows:

#### Image mosaic

Image mosaicking is a tool for splicing multiple adjacent image maps into a large-scale image with spatial connection due to the limitation of the area of remote sensing image maps. The methods of cutting lines and feathering are often used for boundary processing. After multiple adjacent images across the state, the images need to be stitched together using the mosaic tool. The whole process of image mosaic technology includes image preprocessing, image registration, establishment of transformation model, unified coordinate transformation, fusion, and reconstruction.

#### Image cropping

Due to the difference between the directly acquired data space range and the actual range of the research object, when conducting regional research, image cropping is often used to extract the specified range of the original image. This paper mainly uses the mask extraction tool to extract various types of data within the research area data, so that the spatial extent of different data layers after processing is consistent.

#### Resampling

Resampling is the use of ArcGIS to process existing raster data to convert its resolution to a specified size. Since the delineation of ecological space involves a lot of index data, it is necessary to use the resampling tool in the data management tool to unify it into a grid with a resolution of 100 m × 100 m, and then perform overlay calculation.

#### Reclassification

Reclassification is to reclassify and assign values according to the required sequence according to the size of the original data values, so as to obtain a new set of data. Cover the original values of indicators, etc., and use the reclassification in the spatial analysis module to reclassify the indicator data interval and assign sensitive values.

#### Neighborhood analysis

**FIGURE 1:**
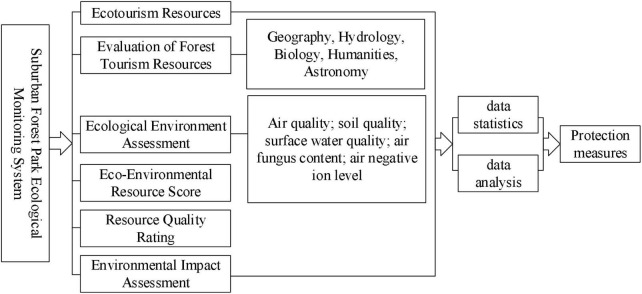
Ecological environment monitoring system diagram.

In Figure 1, re-classification is performed according to the size of the original value, the data set and the original value are obtained, and the re-classification in the spatial analysis module is used to re classify the index data interval and allocate the sensitive value. Since the prior art cannot directly obtain the topographic relief related to the topographic factors, it is necessary to select focus statistics in the neighborhood analysis module of ArcGIS spatial analysis through DEM elevation data, and select 3 × 3 pixels to extract the maximum and minimum values. Raster calculation. There are many forms of data organization and expression of digital elevation model, including regular rectangular grid and irregular triangular grid, which are commonly used in land use engineering.

#### Grid calculation

Using the grid calculator, according to the evaluation model formula of ecological space delineation, the multi-raster layer data is substituted and the superposition operation is performed to obtain the spatial distribution relationship between the evaluation factors.

#### Fuzzy classification

In the evaluation of the importance of ecological service functions, the evaluation results of each subsystem need to be normalized. Select the fuzzy classification in the superposition analysis, input the original evaluation value, select the classification value type as a linear function, and assign the data membership between 0 and 1. The core of fuzzy set is the determination of membership function, which has a great impact on the application effect of fuzzy set. The process of determining the membership function is closely related to the practical application background, and there is no general method.

#### Interpolation analysis

Interpolation analysis is by inputting coordinate points with specific values, and using kriging interpolation or inverse distance weighting (IDW) in the interpolation analysis of the spatial analysis module to assign spatial evolution trends to point coordinate values. Due to the spatial evolution of meteorological station data, it is manifested as the spatial distribution trend of meteorological data such as rainfall and wind speed.

### Ecological environment monitoring system

The construction of the ecological environment monitoring system can comprehensively understand the resources of suburban forest parks, promote the integrity of forest eco-tourism resources, enable forest tourism resources to achieve the most scientific and effective development and utilization, convert various benefits into value, and improve the forest resources. Quality and value of park ecotourism resources.

The main function of the ecological environment monitoring and evaluation system is to confirm the ecological level of the suburban forest park, reduce the adverse impact, and propose reasonable ecological protection measures. Take the ecological environment impact assessment work as a routine work and put it into the various links of the ecological tourism development and construction of suburban forest parks. The research contents mainly include: ecological environment assessment and environmental impact assessment reflecting changes in environmental quality; graded assessment of scenic resource quality and put forward a scientific and effective resource development model. Monitoring should not only ensure systematization and timeliness, but also clearly reflect the existing problems in suburban forest parks, so that effective protection measures can be taken more quickly, so as to continuously optimize the planning and construction management of suburban forest parks, and solve the problems of tourism resources and environment protection issues.

### Evaluation system of ecotourism resources

**FIGURE 2:**
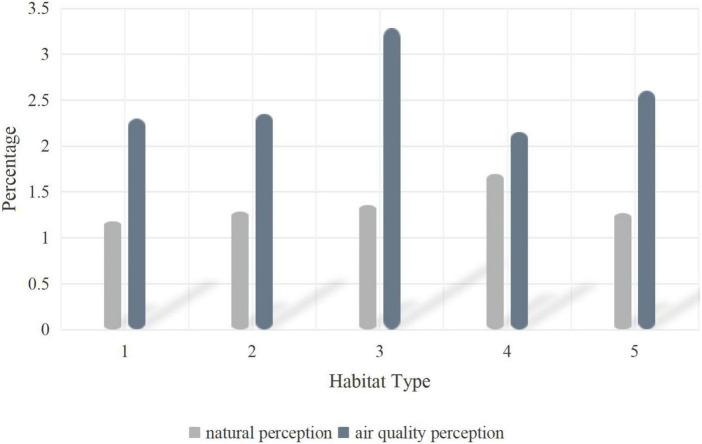
Analysis of differences in natural perception and air quality perception of different habitat types.

**FIGURE 3:**
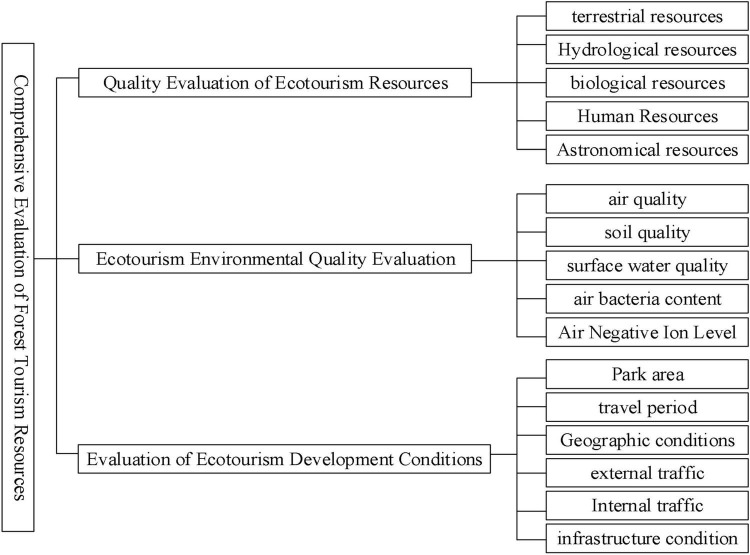
Evaluation framework of ecotourism resources.

In Figure 2, different habitats are analyzed, and reasonable ecological protection measures are proposed through differences in natural perception and air quality to enhance the role of forest resources. In order to give full play to the resource advantages of eco-tourism in the National Forest Park and realize the coordination and unity of various resource protection and planning and construction, the existing natural resources in the National Forest Park from water resources, climate resources, dynamic plant resources, etc. The comprehensive evaluation system of eco-tourism resources is systematically constructed from three directions. Combined with the specific conditions of national forest parks, it is further subdivided into two-level evaluation indicators such as biological resource quality, environmental resource quality, and regional and location conditions. The Delphi method, the expert evaluation method, is mainly used, and the research foundation and practical experience accumulated by experts in ecotourism planning are used to judge the grades of ecotourism resources at all levels, and then the national forest parks are classified according to the evaluation standards of individual tourism resources. The ecotourism landscape resources are classified (see Figure 3).

## Experiment analysis

### Comprehensive ecological characteristic analysis

**FIGURE 4:**
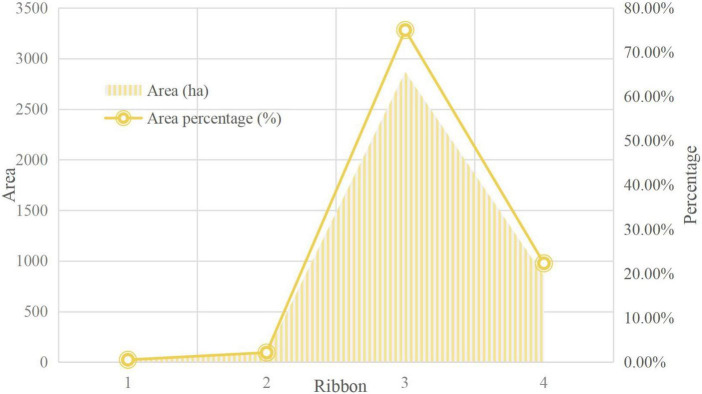
Analysis of functional zoning of forest park.

The division of ecological function zones is mainly based on the types of the ecosystems of suburban forest parks and the differences in ecological functions. In Figure 4, according to the analysis of the functional area division of the forest park, in order to realize the coordination of elimination protection and planning and construction, it is necessary to make full use of the impact of Ecotourism of the National Forest Park. In Table 1, differences in ecological functions are shown. For suburban forest parks, functional zoning can control tourists’ tour routes, and more importantly, protect ecologically sensitive areas, which play a key role in the sustainable development of regional ecosystems and the comprehensive improvement of the ecological environment.

**TABLE 1:** Comprehensive ecological characteristics of different ecological sensitivities.

Ecological grade	1	2	3
Category	Basic suitable land	Suitable land	Very suitable
Land use direction	Restricted planning	Proper planning	Unrestricted planning
Ecological sensitivity	Moderately sensitive	Mildly sensitive	insensitive
Comprehensive ecological characteristics	The terrain is low, the undulations are small, and it is far away from the ecological conservation area.	Mostly agricultural and forestry land	The terrain is low and flat, with abundant tourist attractions and good infrastructure.

### Perception differences of different habitat types

In Table 2, it can be seen that the structure of forest recreation space plays a leading role in improving air quality, and coniferous forests have a more significant improvement in air quality than broad-leaved forests. Samples of lower habitat quality confirmed these patterns, but users in higher quality habitats were more aware of the natural habitat itself.

**TABLE 2:** Differences in nature perception and air quality perception in different habitat types.

	Habitat type	Natural perception	Air quality perception
1	Deciduous broad-leaved forest	1.179	2.295
2	Coniferous and broadleaf mixed forest	1.285	2.349
3	Artificial coniferous forest	1.357	3.286
4	Primeval coniferous pine forest	1.697	2.152
5	Other coniferous forests	1.267	2.600

The results show that aesthetic perception, emotion and natural perception are important factors affecting the cognitive mode of habitat space; the perception of soundscape is driven by the wet habitat; the recognition of the specific parts of Korean pine promotes the user’s perception of the educational function of the habitat; Historic landscape sketches will evoke public perception of historical heritage.

### Functional division of forest park

The details are shown in Table 3.

**TABLE 3:** Functional zoning table of forest park.

Serial number	Ribbon	Area (ha)	The main function	Area percentage (%)
1	Manage service area	20.11	Reception service, customer distribution, administrative management	0.53%
2	Core landscape area	82.73	Ecological tourism, cultural tourism, religious pilgrimage	2.16%
3	General recreation area	2867.24	Ecotourism, forest health, religious pilgrimage	75.00%
4	Ecological conservation area	852.92	Water conservation, soil, and water conservation, ecological maintenance	22.31%

The total planned area of the forest park is 3,823 ha.

As can be seen from the above picture, as a strictly protected area, the ecological conservation area basically does not conduct scenic spot development and resource mining, nor is it open to tourists. The total area is 852.92 ha, accounting for 22.31% of the total area of the forest park. The Forest Park Ecological Conservation Area is strictly in accordance with the regulations, and there are no tourist attractions open to tourists, so that the ecology of the ecological conservation area can develop sustainably and healthily.

## Conclusion

Eco-tourism design planning must be combined with management to improve the management level, clarify the development of forest parks and the short-term and long-term planning and design. Only good design combined with excellent management methods can maximize the benefits of ecotourism; reduce human interference to forest parks. For areas that have been damaged by humans, ecological restoration and reconstruction measures are taken to reconstruct the rich forest ecological landscape structure. At the same time, try to concentrate and narrow the scope of the construction area, pay attention to the protection of ecologically sensitive areas, and maintain the balance of the ecological system in the ecological conservation area; improve the risk awareness of tourists, and avoid the risk of sudden natural or man-made tourism disasters. Implement the strategy of ecological sustainable development of tourism, increase planning and publicity, attach importance to the shaping of tourism products with tourism image and characteristics of suburban forest parks, and expand the tourism market; it is necessary to mobilize the subjective initiative of residents in scenic spots to participate in tourism and promote local economic development, not only considering immediate interests, but also focus on long-term development.

## Data availability statement

The original contributions presented in this study are included in the article/supplementary material, further inquiries can be directed to the corresponding author/s.

## Author contributions

JZ was the experimental designer and the executive of the experimental research of this research. JS completed the data analysis and wrote the first draft of the manuscript. Both authors contributed to the article and approved the submitted version.
